# Perspectives on Development of Measures to Estimate Career Blast Exposure History in Service Members and Veterans

**DOI:** 10.3389/fneur.2022.835752

**Published:** 2022-04-06

**Authors:** Stephanie M. Turner, Stephanie S. Sloley, Jason M. Bailie, Ida Babakhanyan, Emma Gregory

**Affiliations:** ^1^Traumatic Brain Injury Center of Excellence, Defense Health Agency, Silver Spring, MD, United States; ^2^General Dynamics Information Technology, Silver Spring, MD, United States; ^3^Naval Hospital Camp Pendleton, Camp Pendleton, CA, United States

**Keywords:** low-level blast, career exposure, weapons systems, brain health, military

## Abstract

The Department of Defense (DOD) has recently prioritized the investigation of the acute and chronic adverse brain health and performance effects of low-level blast (LLB) generated by the use of weapons systems. While acute exposure can be quantified by sensor technology, career exposure has no widely accepted and validated measure for characterization. Currently, distinct research groups are developing and validating four promising measures to estimate career blast exposure history: the Salisbury Blast Interview, Blast Exposure Threshold Survey, Blast Ordnance and Occupational Exposure Measure, and the Blast Frequency and Symptom Severity. Each measure offers an assessment of blast history that is uniquely beneficial to addressing specific research questions. However, use of divergent strategies is not efficient to accelerate the field's understanding of the impact of career exposure and Service-connected health outcomes. As a DOD-wide solution, collaboration across these groups is required to develop a tool(s) that can be standardized across research studies and, ultimately, pared down to be implemented in clinical settings. Here, we overview the current four measures and provide a perspective on the way forward for optimization and/or combination in support of this solution.

## Introduction

Mild traumatic brain injury (mTBI) has been a priority of the US Department of Defense (DOD) for nearly two decades (1). However, there is recent recognition of hazards to the brain beyond mTBI that may produce cognitive and physical alterations, potentially impacting the strength and readiness of the force (2). One of these hazards is low-level blast (LLB) exposure (i.e., relatively predictable overpressure exposure from outgoing munitions) (3). For acute LLB exposures, the use of blast sensors during training events is beneficial for the direct quantification of discrete blast characteristics (e.g., peak overpressure, impulse, etc.). The objective data collected from these sensors can be related to acute symptomology and other Warfighter health and performance metrics to begin to understand and model acute dose-response relationships. However, these sensors are limited to prospective, time-limited data collection in discrete training events among a small, targeted group of at-risk Service members (SM) (e.g., 10-day explosive entry training course participants) (4, 5). To expand our understanding of the impact of LLB exposure on Service member and Veteran (SMV) brain health, there is a need to reliably and accurately characterize career-long exposures. The most viable approach to prospective data collection is the development of self-report measures designed to capture career LLB exposure. Despite the subjective nature of self-report measures, this data collection is the only approach that can feasibly and economically estimate retrospective exposures across large SMV populations. Until the research field can widely implement a validated self-report measure, pertinent and accurate exposure history for up to 20+ years of service will remain undocumented. Accordingly, the field will be limited in its ability to synthesize findings across studies investigating SMV long-term brain health.

Recently, the lack of a validated, self-report measure to characterize career LLB exposure has been identified in multiple National Defense Authorization Acts with a specific call for documentation of blast exposure history in the SM medical record (6). Aligned to these requirements, a number of research groups are developing and validating various methods that can be used for estimation of career LLB. These surveys and analytical approaches are in a nascent stage, with little available published data on the psychometric properties or the utility in research environments. Consequently, some of these novel measures may overlap in assessing similar domains (e.g., categories of weapons, symptomology) but may do so using differing approaches that limit their comparability and potential to measure important factors relevant to SMV brain health. This paper reviews the following key measures currently being developed to characterize blast: the Salisbury Blast Interview (SBI), Blast Exposure Threshold Survey (BETS), Blast Ordnance and Occupational Exposure Measure (BOOM), and the Blast Frequency and Symptom Severity (B-FASS) (Table 1). Based on the description of these measures, we provide perspectives to assist in their validation, utilization in research studies, and future application to clinical settings.

**Table 1:** Summary of measures utilized to capture career exposure.

**Measure**	**Purpose**	**Question format**	**Time to complete**	**Weapons use**	**Primary outcome measure**
SBI (7)	To characterize career blast or explosion exposure	Structured interview	10–30 min	Weapon identification (limited list) as the source of blast exposure	Characterization of individual experience of career blast event and the associated environmental and blast characteristics
BETS (8)	To identify and characterize likelihood of symptoms associated with lifetime exposure to LLB specifically from weapons systems categories	Online survey	5–8 min	Calculated frequency of use across each of 5 weapons categories	GBEV, determination of exposure thresholds at which an individual is likely to experience certain symptoms
BOOM (9)	To provide estimated exposure, operational and training-based, across weapons systems	Paper and pencil survey	5–10 min	Calculated total exposure number across weapons systems	Characterization of exposure and source of exposure in specific clinical populations
B-FASS (10)	To quantify the frequency and characterize the symptomology of blast exposures, including from individual weapons systems	Semi-structured (adaptive) survey	Approximately 5 min	Level of experience with individual weapons systems	Characterization of career blast-related symptomology (frequency and duration)

*BETS, Blast Exposure Threshold Survey; B-FASS, Blast Frequency and Symptom Severity; BOOM, Blast Ordnance and Occupational Exposure Measure; GBEV, Generalized Blast Exposure Value; LLB, low-level blast; SB, Salisbury Blast Interview*.

## Self-Report Measures of Career Blast

### SBI

The SBI, designed at the W. G. (Bill) Hefner VA Healthcare System, is unique from the other developed measures as it is a structured interview rather than a self-report questionnaire. It is intended to be completed in 10–30 min and provides a characterization of blast exposure across the lifespan (7). The interview queries any and all exposures to blast sustained in military service, to include those exposures that occurred during combat. For each experience regardless of blast source, the interview details specific variables about the blast event including: date of event, personal protective equipment (PPE) present, effects of blast, individual experience of blast characteristics (i.e., wind, debris, ground shaking, pressure change, temperature change, sound), and distance from event. It also queries the source of the blast exposure using a discrete list (i.e., mortar, rocket, improvised explosive device [IED], grenade, rocket-propelled grenade, missile, bomb, landmine, other) which extends recorded exposures beyond LLB to more adequately capture the details of high-level blast. To reduce time burden, the SBI includes a mechanism to evaluate highly similar events in which a representative event is described along with the number and time frame for all similar events. The interview produces a rich dataset about these experiences and can provide critical, contextual information about key blast exposure characteristics. This tool has been used to understand risk for psychiatric and cognitive symptoms as well as neuroimaging alterations in combat veterans across the full spectrum of blast exposures (11–14). However, the time and administration requirements may make the measure more challenging to include in large-scale research studies in comparison to self-report questionnaires. More research is needed to determine if the structured interview adds additional value in comparison to self-report questionnaires.

### BETS

Unlike the structured interview format of the SBI, the BETS is a 5–8 min survey designed by Naval Medical Research Center and Walter Reed Army Institute of Research to describe the likelihood of developing symptoms (questions adapted from established post-concussive symptom questionnaires) associated with lifetime exposure to blast from weapons systems (15, 16). The BETS does this by first querying the number of times the respondent has been exposed to the following five operationally-defined categories of weapons: (Category 1) small to medium firearms; (Category 2) shoulder-mounted firearms or large arms that can be carried by a person; (Category 3) artillery, missile weapon systems, or large arms carried by a vehicle; (Category 4) smaller explosives, grenades and IEDs; and (Category 5) larger explosives, targeted explosives in close range, and non-military occupational explosives. The responses to the BETS have been used to develop the primary outcome measure referred to as the Generalized Blast Exposure Value (GBEV), a weighted numerical value that represents the units of blast exposure over a lifetime. The GBEV has not been validated but may have potential to identify individuals at an elevated risk for symptomology after cumulative exposure. This survey was recently characterized in a sample of SMs (*n* = 984) (8) and has since been implemented by non-affiliated research groups (i.e., Defense and Veterans Brain Injury Center-Traumatic Brain Injury Center of Excellence 15-Year Longitudinal TBI Study; Long-Term Impact of Military-Relevant Brain Injury Consortium Chronic Effects of Neurotrauma Consortium; CARE-SALTOS Integrated Study administrated by the National Collegiate Athletic Association-DOD Grand Alliance Concussion Assessment, Research and Education Consortium) to capture lifetime blast exposure.

### BOOM

The National Intrepid Center of Excellence is performing a multi-phase development process to produce the BOOM, a 5–10 min survey capturing career blast exposure. The BOOM is a partial adaptation of the BETS (9) but designed with intended use in clinical settings. The BOOM estimates total number of exposures in a career, distance from blast, military setting (i.e., training or operational), and exposure origins (i.e., incoming or outgoing munitions). To query weapons use, the BOOM incorporates Categories 2 and 3 from the BETS and adapts Categories 4 and 5 by subdividing them to specifically probe explosive breaching, explosive ordnance disposal (EOD), and IEDs; small to medium firearms (Category 1) were determined to be less clinically relevant for their patient population than the other weapon sources. The BOOM has undergone evaluation to optimize and gain insight into the content and administration procedures required for the intended clinical environment. Currently, an interdisciplinary research team is developing a study to determine the psychometric properties of the BOOM and investigate associations with long-term health outcomes in targeted patient populations. The findings from this validation study will facilitate future use in clinical and research settings in specialty clinics across the Military Health System.

### B-FASS

Developed by researchers at Walter Reed National Military Medical Center, the B-FASS is an adaptive, tablet-based survey that aims to characterize the symptomology of career blast exposures, including those from individual weapons systems (10). It first asks respondents to report the number of career blasts experienced (i.e., never, one time, two times, 3–5 times, 6–9 times, tens of times [10–99], hundreds of times [100–999], thousands of times [1,000+]) and how many of those blast exposures were at close enough distance to feel the heat or pressure of the blast. The survey then titrates subsequent questions to query symptom provocation associated with those exposures and the weapons system believed to be the cause of the most severe symptoms. The questions emphasize the number of times a specific symptom was experienced, and the length of time the symptom persisted (e.g., from a few seconds to >3 months). To minimize testing burden, respondents are first asked about their experience with general categories of weapons (e.g., shoulder-mounted firearms) and common blast-related symptoms (e.g., sensory). Respondents are only asked detailed follow-up questions regarding specific weapons (e.g., light anti-tank weapon) or symptoms (e.g., hearing difficulty and sensitivity to noise) for the endorsed weapons categories. The data collected via this survey (on over 2,000 SMs) have not yet been published, as this assessment is still being evaluated and optimized based on end-user feedback to improve the data collection process.

## Comparison Across Measures

Given the relationship between high-level blast and mTBI, these kinds of exposures have been more widely studied, but the investigation of LLB as a hazard to the brain has become an important research priority for the DOD. Each of the measures discussed here has the ability to characterize career high and low-level blast exposures, but the SBI uniquely captures details of high-level blast exposures, while the BETS, BOOM, and B-FASS more specifically estimate career LLB from weapons systems. To understand the utility of these tools in LLB-related research and potential implementation in clinical practice, we consider two constructs that allow for comparison of these measures: (1) estimation of LLB exposure and (2) the relation of LLB exposure to symptomology and other outcome measures.

### Estimation of LLB Exposure

To capture career LLB, a measure needs to query, at a minimum, the number or frequency of exposures and the alignment of those exposure to weapons use. Although the SBI arrives at a numeric estimation of career exposures, it does not specifically evaluate exposure to weapons. In this way, it does not provide sufficient granularity to evaluate the number of LLB events generated by specific weapons categories. Unlike SBI, the B-FASS does numerically estimate career exposures. Subsequently, the B-FASS asks about both general categories of weapons and followed by questions related to individual weapons systems for any endorsed categories, but the focus of these questions is associated symptomology. As a result, B-FASS cannot attribute the career exposures to specific weapons categories or systems.

BETS and BOOM both estimate total number of career blast exposures as well as number of LLB exposures to specific categories of weapons. Therefore, BETS and BOOM are most applicable for estimating career blast and career LLB. Given the inclusion of small and large IEDs and EOD as well as the lack of specificity to the setting of these exposures, these two measures also have the potential to capture some less predictable and higher level exposures alongside the LLB exposures.

Although BETS and BOOM are currently the optimal measures for estimating number of weapons-related career LLB exposures, there are factors utilized by the other measures that warrant further investigation. From a mathematical approach to estimate number or frequency of career blast exposures, BETS uses a time-based equation (e.g., number of rounds per day, days per week, etc.) to estimate the number of exposures from specific weapons categories whereas B-FASS simply asks respondents to select from ranges of exposures. There is currently no data supporting the validity of either approach. With regard to characterizing career LLB exposure by weapons use, it is unknown whether categories or individual systems are most relevant, whether any of these measures are using the optimal categorization of weapons, and whether there is a differential effect of individual weapons systems within a single category on brain health and performance outcomes. As this field of research answers the above questions, the specificity utilized in a measure may be adapted to fit the research question or clinical outcome of interest.

### Relation of LLB Exposure to Symptomology and Other Outcomes

The level of inquiry into symptoms related to LLB exposures differs between the measures. While the BETS and BOOM are most specific to LLB characterization, they are not designed to capture detailed symptom information. The BETS determines the likelihood of symptomology associated with exposure but without detail on specific symptoms and symptom duration. Conversely, the BOOM estimates blast exposure without probing symptomology, though the developers plan to examine relationships of exposure captured by the measure with concurrent symptomology data collected via other self-report tools. Similarly, exposure-related symptomology is not collected with the SBI as it has been used with established self-report symptom inventories. The most detailed query into blast-related symptomology can be accomplished with the B-FASS, which asks about the number of exposures that elicited a specific acute symptom as well as the duration of symptom persistence following exposure. Additionally, B-FASS was designed to be compared with objective audiometric data allowing for a multifaceted understanding health outcomes related to blast.

## Discussion

In response to the Congressional mandate requiring documentation of blast exposure history, efforts are underway to move such information collected from overpressure sensors into the SM medical record. However, as previously mentioned, blast sensors are relevant for capturing only prospective, time-limited data from discrete exposures which limits associations to only acute health and performance outcomes. Sensor data are incapable of directly informing more chronic health issues, yet a DOD-wide method to capture individual chronic exposure does not exist. A self-report measure designed to capture career LLB better addresses chronic exposure and, as an adjunct to sensor data, can present a more holistic understanding of LLB in relation to health outcomes and any Service-connected dysfunctions that may develop.

It is important to note that other methods for estimating LLB exposure do exist and do serve other, complementary purposes. First, epidemiological investigations have used military occupational specialty (MOS) as a proxy for estimating cumulative or career exposure (17, 18). Though this approach can be informative, especially when used to query large datasets or in conjunction with one of the aforementioned prospective measures, it does not reflect the large amount of variability between individuals and exposures within the same MOS. Second, the primary method for estimating blast in relation to injury has been leveraging structured interviews that are explicitly designed to capture TBI. These interviews (e.g., Boston Assessment of TBI – Lifetime, the Virginia Commonwealth University Retrospective Concussion Diagnostic Interview - Blast, the Ohio State University Traumatic Brain Injury Identification Method, the Minnesota Blast Exposure Screening Tool) often relate blast to key outcomes (e.g., post-traumatic amnesia, loss of consciousness, altered mental status, and symptomology) to identify instances of probable TBI. Since LLB exposures traditionally do not result in signs or symptoms that rise to the level of clinically diagnosable TBI, the sensitivity of these TBI measures for blast exposures without the aforementioned outcomes is relatively low. Although MOS and these interviews can provide some overview of the influence of blast exposure and the relationship of blast and health outcomes or injury, measures specifically capturing career LLB serve an important role in documenting a comprehensive blast exposure history.

Given the requirement for a career blast exposure measure that assess LLB, it is not sustainable or efficient for research groups to continue to pursue measure development and validation efforts in isolation. Moving forward, collaboration across US DOD partners is required to produce a standardized approach for capturing such exposure. Each of the currently developed or developing measures offers critical information that has previously not been captured with respect to LLB; however, uncoordinated lines of inquiry into these various measures limits their use across the broader research and clinical fields and obfuscate the best way forward for capturing career LLB exposure. Rather, streamlined metric development, drawing on the expertise from individuals across these research groups, and subsequent validation studies with samples representative of the Armed Forces offers improved applicability and implementation within the DOD. To this end, the establishment of a working group with experts integral to the development of each of these measures of career blast exposure is required. This working group can determine for research and/or clinical populations whether the best course of action is to: a) optimize one of the current measures, or b) combine aspects from each of the current measures to produce a new tool more suited to implementation within the DOD. Key considerations include a consensus on the operational definition of LLB for standard implementation in a DOD-wide tool, required specificity to weapons categories and/or individual weapons systems, and mathematical approach for estimation of LLB. Researchers and clinicians should be included in this group to advise on the current or potential usability as well as to differentiate the requirements of the ideal measure(s) for their respective populations. Specifically, the logistics (e.g., appointment lengths) and decision making requirements (e.g., course of treatment, specialty referral) of the clinical setting offer a unique set of constraints that are often not present in research settings and likely will necessitate different end products. Until the ideal tool(s) is identified, there will continue to be a limited understanding of the effects of LLB on brain health with respect to individual factors (e.g., TBI or high-level blast exposure history), symptomology (e.g., incidence, severity, and duration of most prevalent symptoms), and clinical complaints (e.g., medical diagnoses and healthcare utilization) among SMVs.

## Data Availability Statement

The original contributions presented in the study are included in the article/supplementary material, further inquiries can be directed to the corresponding author/s.

## Author Contributions

ST and SS led the effort. EG contributed to the design of the effort. JB and IB contributed to the framework of the article. All authors contributed to the development of the manuscript, provided comments, and revisions.

## Funding

This work was supported by the Traumatic Brain Injury Center of Excellence.

## Author Disclaimer

The views expressed in this manuscript are those of the authors and do not necessarily represent the official policy or position of the Defense Health Agency, Department of Defense, or any other U.S. government agency. This work was prepared under Contract HT0014-21-C-0012 with DHA Contracting Office (CO-NCR) HT0014 and, therefore, is defined as U.S. Government work under Title 17 U.S.C.§101. Per Title 17 U.S.C.§105, copyright protection is not available for any work of the U.S. Government. For more information, please contact dha.TBICOEinfo@mail.mil.

## Conflict of Interest

ST, SS, JB, and IB were employed by General Dynamics Information Technology. The remaining author declares that the research was conducted in the absence of any commercial or financial relationships that could be construed as a potential conflict of interest.

## Publisher's Note

All claims expressed in this article are solely those of the authors and do not necessarily represent those of their affiliated organizations, or those of the publisher, the editors and the reviewers. Any product that may be evaluated in this article, or claim that may be made by its manufacturer, is not guaranteed or endorsed by the publisher.
